# Lipid content and stable isotopes of zooplankton during five winters around the northern Antarctic Peninsula

**DOI:** 10.1038/s41597-020-00722-9

**Published:** 2020-11-11

**Authors:** Jennifer Walsh, Christian Reiss

**Affiliations:** Antarctic Ecosystem Research Division, Southwest Fisheries Science Center, National Marine Fisheries Service, National Oceanic and Atmospheric Administration, La Jolla, CA 92037 USA

**Keywords:** Stable isotope analysis, Lipids, Marine biology, Ecosystem ecology

## Abstract

The Southern Ocean zooplankton community is diverse, yet most species are understudied, especially with respect to their overwinter feeding ecologies. Here we present body condition and trophic biomarker data (lipid content and stable isotopes of carbon and nitrogen) from 19 zooplankton species collected over five consecutive winters (August and September 2012–2016) around the northern Antarctic Peninsula. We report environmental data (percent sea-ice cover, sea-ice type, water temperature, salinity, and integrated chl-a) as well as species abundance data at each sampling location to provide additional context for interpreting the lipid and stable isotope data. For most species, these are the first winter measurements or time series of body condition, trophic position, and abundance in relation to environmental variables. These data are critical for evaluating changes in ecosystem structure and predator-prey relationships in a region of Antarctica that is warming faster than most other areas on Earth as a result of climate change.

## Abstract (summary)

**Table:** 

Measurement(s)	lipid • stable isotope analysis • concentration of chlorophyll a in water • sea ice • species abundance • daylight conditions • carbon content • nitrogen content • temperature of water • ocean salinity
Technology Type(s)	chemical extraction • elemental analysis isotope ratio mass spectrometry • fluorometer • visual observation method • Count • environmental condition • Temperature Sensor Device • Sensor Device
Factor Type(s)	water-column chlorophyll-a
Sample Characteristic - Organism	Amphipoda • Copepoda • Chaetognatha • Polychaeta • Cnidarian • Ctenophora • Euphausiidae • Gastropoda • Tunicata
Sample Characteristic - Environment	ocean
Sample Characteristic - Location	West Antarctica

Machine-accessible metadata file describing the reported data: 10.6084/m9.figshare.13003151

## Background & Summary

Of all zooplankton species in the Southern Ocean, Antarctic krill (*Euphausia superba*) is by far the most studied. However, several other zooplankton species play important roles in the Southern Ocean carbon cycle as grazers^1–3^, prey for fishes, seabirds, and other zooplankton^4–8^, and as symbiotic parasites^6,9^. These species are poorly understood compared to *E. superba*, especially with respect to their overwinter feeding ecologies. As sea-ice extent and duration along the western and northern Antarctic Peninsula decline because of regional warming^10,11^, concomitant changes in primary productivity may result in shifts in the structure of the zooplankton community^12–14^. These shifts in species composition and distribution may have cascading effects on the food web, with potentially negative consequences for higher trophic levels^14^.

Indicators of body condition and trophic position in zooplankton may be useful proxies for evaluating the overall health of an ecosystem. Lipid content can be used as an indicator of body condition, with higher lipid content reflecting better body condition and a more favorable feeding environment (as demonstrated in Antarctic krill^15^). Stable isotopes of carbon (^13^C/^12^C, denoted as δ^13^C) reflect the provenance of carbon in prey sources, with benthic and ice-associated sources being more enriched in δ^13^C than pelagic and open-water sources^16–18^. Stable isotopes of nitrogen (^15^N/^14^N, denoted as δ^15^N) reflect the trophic level of the consumer, with each successive level enriched in δ^15^N by 2‰–3‰^3^. Around the northern Antarctic Peninsula, where seasonal sea ice is dynamic and unpredictable, these proxies, considered in concert with the environmental conditions at the time of sampling (e.g., sea-ice cover as an indicator of resource availability in the ice and chlorophyll-a as an indicator of resource availability in the water column), provide valuable information for inferring ecosystem status and how populations of different zooplankton species may respond to increased warming.

**Fig. 1:**
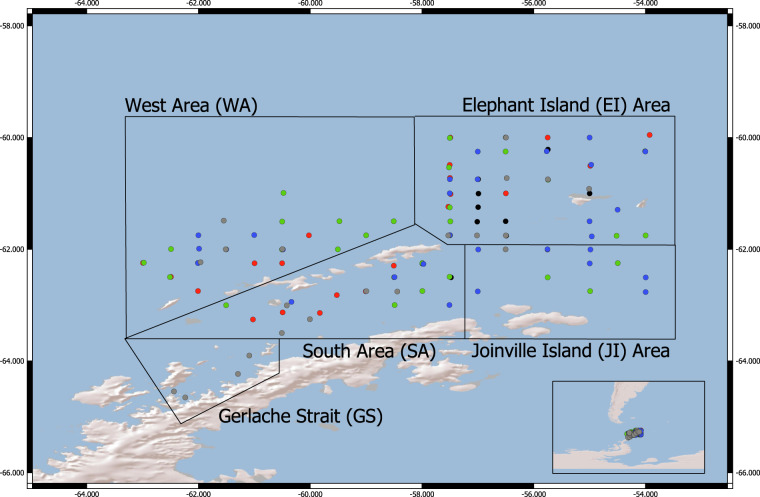
The U.S. Antarctic Marine Living Resources (AMLR) Program’s survey area in the northern Antarctic Peninsula. The inset in the lower right corner shows the survey area relative to the Antarctic and South American continents. Dots indicate sampling stations where zooplankton were collected for both lipid content and stable isotopes, colored by year: black: 2012; red: 2013; green: 2014; blue: 2015; grey: 2016. Specific locations for each sample are provided in the dataset, while sample sizes are provided in Online-only Table 1.

These samples were collected by the U.S. Antarctic Marine Living Resources (AMLR) Program during five winter surveys around the northern Antarctic Peninsula and the South Shetland Islands (Fig. 1). The primary goal of these surveys was to estimate the biomass of Antarctic krill using acoustic and net sampling techniques; however, other zooplankton species were collected opportunistically and analyzed when time permitted (Online-only Table 1). Although not all species were sampled each year and, in some cases, the numbers of samples are small, many of these measurements are the first of lipid content and stable isotopes for several species in winter. We believe these data are an important contribution to the growing number of winter ecosystem studies in Antarctica and may help future researchers interpret similar data in the context of a changing environment.

## Methods

### Survey area

The U.S. AMLR Program conducted five winter surveys (August and September 2012–2016) around the northern Antarctic Peninsula aboard the U.S. National Science Foundation research vessel/ice breaker (RVIB) *Nathaniel B. Palmer* (Fig. 1)^19^. We surveyed a historical grid of 110 fixed stations located 20–40 km apart around the northern Antarctic Peninsula and South Shetland Islands, which was divided into four sampling areas: the Elephant Island Area (EI; 43,865 km^2^), the South Area (the Bransfield Strait, SA; 24,479 km^2^), the West Area (the west shelf immediately north of Livingston and King George Islands, WA; 38,524 km^2^), and the Joinville Island Area (JI; 18,151 km^2^). In 2016, we also surveyed the Gerlache Strait (GS; 24,479 km^2^).

### At-sea sampling

Detailed methods for all at-sea sampling by the U.S. AMLR Program have been previously reported^19^. At each sampling station, we performed a Conductivity-Temperature-Depth (CTD) cast to 750 m or to within 10 m of the bottom in shallower areas (SBE9/11; Sea-Bird Electronics). The CTD rosette was equipped with 24 10 l Niskin bottles triggered to close on the upcast at 750, 200, 100, 75, 50, 40, 30, 20, 15, and 5 m. We defined the Upper Mixed Layer (UML) depth (m) as the depth at which the density of the water changed by 0.05 kg m^−3^ relative to the mean density of the upper 10 m of the water column^20^. UML temperature and salinities were defined to be means over the depth range of the UML. We defined daylight conditions according to three categories. Day (D) was defined as one hour after local sunrise to one hour before local sunset; night (N) was defined as one hour after sunset to one hour before sunrise; and Twilight (T) was defined as one hour before and after sunrise and sunset.

Chlorophyll-a (hereafter chl-a) was determined from water samples collected between 5 and 200 m^21,22^. Samples (285 ml) were filtered at a pressure differential of < 150 mm Hg through 25.4 mm glass fiber filters (Whatman, Inc.), and chl-a was extracted from filters in 7 ml methanol over 24 hrs. Samples were then centrifuged and chl-a was determined from the recovered supernatant using a Trilogy fluorometer (Turner Designs). Samples were then acidified using 2 drops 1.0 N HCl and read again to determine phaeopigment concentration. We calculated integrated chl-a (to 100 m, mg chl-a m^−2^) using concentrations at the discrete depths of each sample from 5 m to 100 m and averaging between depths to calculate the intermediate depth concentrations. Concentrations were then summed over the top 100 m for each CTD cast as an indicator of water-column food availability.

At each station, we towed an Isaacs-Kidd Midwater Trawl (IKMT) net with 505 µm mesh to 170 m or to within 10 m of the bottom in shallower areas. The volume of seawater filtered by each tow was determined using a calibrated flow meter mounted on the net frame (Model 2030 R, General Oceanics, Inc.). Large zooplankton (identifiable without a microscope) were enumerated and identified to species. Small zooplankton (requiring a microscope for identification) were subsampled by rinsing the cod end of the net into a 505 µm sieve and suspending the recovered zooplankton in a known volume of ambient seawater. The water was thoroughly mixed and zooplankton in 1–2 ml subsamples were placed on Petri dishes and identified under a microscope. At least five subsamples were counted per tow, and total counts of individuals were scaled up to the volume of the tow. Abundance of each species was calculated by dividing the total number of individuals by the tow volume and multiplying by the tow depth.

### Sea ice

Beginning in 2013, we determined percent sea-ice cover and sea-ice type at each sampling station by observing the ice conditions in a 200 m arc around the stern of the ship during each net tow. Percent sea-ice cover was characterized as 0% (open water), 1–25% (recorded as 25%), 26–50% (recorded as 50%), 51–75% (recorded as 75%), and 75–100% (recorded as 100%). Sea-ice type was classified according to a modification of the standardized visual approach from the Scientific Committee on Antarctic Research Antarctic Sea Ice Processes and Climate Program (ASPeCT)^23^. We classified ice type as slush (frazil, shuga, and grease ice; less than 100 mm thick), thin (nilas, pancake, young grey, and young grey-white ice; 100–300 mm thick), first-year (thicker and more solid ice; 300–1200 mm thick), and multi-year (more solid; any ice thicker than 1200 mm).

### Lipid analysis

Individuals of each species collected from the same station were frozen together at −20 °C until lipid analysis (usually no later than 48 hrs after collection). At stations where too few individuals were collected to achieve a mass suitable for lipid analysis (0.5–1.5 g), individuals from multiple stations within the same sampling area were pooled for analysis.

Lipid was extracted from aliquots of 0.5–1.5 g of homogenized animals^24,25^. Samples were refrigerated overnight in a 2:1 chloroform:methanol solution with 0.01% butylated hydroxytoluene (BHT) as a preservative. The following day, samples were filtered to collect the solution, and the lipid-extracted zooplankton were retained in glass vials for stable isotope analysis. The solution was centrifuged for 20 min and the methanol layer was removed and discarded. Lipid dissolved in the chloroform layer was filtered through anhydrous sodium sulfate to remove all traces of remaining methanol and water. The chloroform solvent was then evaporated under nitrogen and lipid was weighed to the nearest 0.001 g (Model PA214, S/N: 8331270072, Ohaus) to determine percent lipid in each sample.

Percent lipid of both wet mass and dry mass are reported in the full dataset. Dry mass of samples was determined by freeze-drying all 2016 lipid-extracted samples in glass vials for 16 hrs (VirTis Benchtop K Lyophilizer, SP Scientific), adding back the mass of the removed lipid, and then subtracting the mass of the vial. The mean percent mass loss for each species was calculated and applied to the wet mass of each sample to determine dry mass.

### Stable isotope analysis

The same samples used for lipid analysis were used for stable isotope analysis and prepared according to the analytical laboratory’s guidelines^26^. Vials of lipid-free zooplankton were freeze-dried for 14–16 hrs. Dried samples were pulverized and 0.8–1.2 mg aliquots were weighed into tin capsules (5 × 9 mm, Costech Analytical Technologies) and analyzed for δ^13^C and δ^15^N at the University of California Davis Stable Isotope Facility (UCD SIF) using a PDZ Europa ANCA-GSL elemental analyzer interfaced to a PDZ Europa 20–20 isotope ratio mass spectrometer (Sercon). Isotope values are reported as ratios of heavy to light isotopes (δ^13^C and δ^15^N) in parts per thousand (‰) compared to standards (PeeDee limestone and atmospheric N_2_, respectively).

Because the zooplankton species analyzed contain few, if any, calcified structures, samples were not acid treated prior to stable isotope analysis to eliminate the risk of skewing δ^15^N values^27^. Lipid removal prior to stable isotope analysis may also skew δ^15^N values. Lipid is often removed because it is more depleted in ^13^C than protein or carbohydrate and may result in inaccurate characterizations of δ^13^C if not removed, particularly in organisms with high lipid content^28^. However, chemical lipid extraction following the same technique we used^24^ may only skew δ^15^N by approximately 0.25‰^28^, which is close to typical analytical error. To address the issue of skewed δ^15^N resulting from lipid extraction, we collected as many extra samples of all zooplankton species as possible in 2015 and 2016 for stable isotope analysis of non-lipid-extracted animals. Those data are included in the dataset and denoted as “WAN” (whole-animal), whereas lipid-extracted samples are denoted as “LFR” (lipid-free). The δ^13^C in whole-animal samples was not normalized for lipid content.

## Data Records

The data from this study are presented in one comma-separated values (csv) file^29^. Each row corresponds to one sample, with environmental and species abundance data for the sampling station.

### Incomplete environmental data

For samples from pooled stations (denoted as “POOLED” in the station column), we estimated tow depth and environmental variables by calculating the mean of each variable for the sampling area and year. Reported abundance for pooled stations is the mean abundance for a given species within the sampling area and year. To estimate sea-ice type, we assigned a number to each ice type (0 = none, 1 = slush, 2 = thin, 3 = first, 4 = multi), calculated the mean for the area and year, and rounded the result to the nearest whole number. Station coordinates and daylight conditions are not reported for samples from pooled stations (denoted as “NA”). For one sample (2013ECR04), daylight conditions were not recorded during sampling; for this station, daylight conditions is denoted as “NR” in the dataset. For another sample (2014TM01), station data were not recorded during sampling; for this station, latitude, longitude, and daylight conditions are denoted as “NR” in the dataset.

In 2012, percent sea-ice cover and sea-ice type were not determined. Sampling stations were characterized as “ice” (100%) or “no ice” (0%). First-year sea ice dominated stations with ice; however, at one station with ice (W05.509), sea-ice type was not recorded and is denoted as “NR” in the dataset. At one other station (W1010), no sea-ice conditions were recorded and both percent sea-ice cover and sea-ice type are denoted as “NR” in the dataset.

### Incomplete lipid and stable isotope records

For most species in 2012, the number of individuals analyzed for lipids and stable isotopes were not recorded (denoted as “NR” in the dataset). In all other years, counts of individuals are recorded except for the smallest animals analyzed (*Metridia gerlachei* and *Limacina helicina*), where counting individuals would have been impractical.

In a few cases, lipid was undetectable in samples. These are denoted “ND” (for none detected) in the dataset. Stable isotope values are reported for these samples.

In some cases, especially for gelatinous species, the calculated percent mass loss when wet samples were dried exceeded 100%. In these cases, only percent lipid of wet mass is reported in the dataset.

For one sample in 2012 (2012TM02), we were unable to obtain stable isotope values. Stable isotopes are reported as “NR” for this sample, but lipid data for this sample are reported.

Numbers of individuals per whole-animal (“WAN”) sample are reported, but no mass or lipid data are reported since the samples were not weighed for lipid analysis and lipid was not extracted from these samples. For one sample (2015ECR06), the number of individuals was not recorded and is denoted as “NR” in the dataset.

### Other notes

During surveys, counts of the siphonophore *Diphyes antarctica* were recorded under the general category of “Siphonophore.” Abundances for *D. antarctica* may therefore reflect other species of siphonophores, although *D. antarctica* was the most common species encountered.

## Technical Validation

### Chlorophyll-a measurements

The fluorometer used for chl-a measurements was calibrated with a chl-a standard (*Anacystis nidulans*, Sigma-Aldrich, Inc.). The standard was resuspended in 90% acetone and the concentration was spectrophotometrically determined using published absorption coefficients^30^ for chlorophyll dissolved in 90% acetone. We then performed a 5-point calibration using methanol as the solvent at the beginning and the end of each survey so that the calibrations and all fluorescence measurements were done using methanol, which has been shown to be a more efficient solvent for extracting pigments from phytoplankton than acetone^22^. Methanol “blanks” and a solid-state standard (Turner Designs) were run at the beginning and end of each day’s sample measurements to ensure accuracy.

### Zooplankton Identification

Several technicians identified zooplankton throughout our 5-year study. We had one lead zooplankton specialist who trained each technician prior to each survey, and two senior zooplankton specialists on all five surveys. These three individuals (the same individuals over the five years) routinely checked the identifications made by all other technicians to ensure consistency. Data from each net tow was tallied by one person, checked by a second person, and re-checked and entered into a database by a third person. After each net tow, the lead zooplankton specialist checked the data in the database to ensure accuracy.

### Lipid analysis

All sample preparation and lipid extraction throughout our 5-year study was performed by one person (the lead author) to reduce error potentially introduced by inconsistencies among multiple laboratory technicians.

Lipid extracted at sea was stored in glass centrifuge tubes that were pre-weighed on an analytical balance that was metrologically tested and calibrated by Mettler-Toledo technical staff at NOAA’s Southwest Fisheries Science Center (SWFSC) prior to surveys. Samples were partially evaporated under nitrogen while at sea, and then flushed with nitrogen, sealed, and stored at −80 °C for transport back to SWFSC. The evaporation process was completed at SWFSC and the pre-weighed tubes were weighed again on the same calibrated balance to ensure consistency and accuracy of lipid masses.

### Stable isotope analysis

The UCD SIF uses at least four different laboratory reference materials, previously calibrated against international reference materials, when analyzing stable isotope samples. Details of their calibration protocol can be found at https://stableisotopefacility.ucdavis.edu/13cand15n.html. Their reported long-term standard deviations for ^13^C and ^15^N are 0.2‰ and 0.3‰, respectively.

In 2012, we analyzed two replicates of 12 samples. The mean difference in δ^13^C was 0.18759 ± 0.0875‰ (SD), and the mean difference in δ^15^N was 0.12685 ± 0.09567‰ (SD). As these values were within instrumental error, replicates were not analyzed in subsequent years.

## Online-only Table

**Online-only Table 1:** Summary of sampled species by taxonomic group, years sampled, and number of samples per year.

Taxonomic group	Species	Years sampled	n per year
Amphipods	Cyllopus magellanicus	2015	2
		2016	2**
	Eusirus propeperdentatus	2016	1
	Eusirus spp.	2014	1
	Primno macropa	2016	2**
	Themisto gaudichaudii	2012	2
		2014	3
		2015	3
		2016	2**
	Vibilia antarctica	2014	2**
		2015	3
		2016	1, 2**
Copepods	Calanus propinquus	2013	1
		2014	1
		2015	1
		2016	1**
	Metridia gerlachei	2013	1
Chaetognaths and polychaetes	Chaetognatha	2014	1, 1**
		2015	3
		2016	1
	Tomopteris spp.	2014	1
		2015	1
Cnidarians (siphonophore)	Diphyes antarctica	2015	3
		2016	1
Ctenophores	Mertensiidae	2015	2
Euphausiids	Euphausia crystallorophias	2012	2*
		2013	6, 1**
		2014	4
		2015	4, 1**
		2016	3, 1**
	Euphausia frigida	2012	6
		2013	8, 2*
		2014	7
		2015	4, 1**
		2016	5
	Euphausia triacantha	2012	1
		2013	5, 2*
		2014	4
		2015	3
		2016	4
	Thysanoessa macrura	2012	6, 2*
		2013	5, 3*
		2014	12
		2015	12
		2016	10
Gastropods	Clione limacina	2014	1, 1**
		2015	2, 1**
		2016	1**
	Limacina helicina	2014	1, 1**
		2016	1
Tunicates	Salpa thompsoni	2014	4, 2*
		2015	4
		2016	2

One asterisk in “n per year” column indicates replicates from the same sampling station (e.g., “8, 2*” indicates that 8 samples were from unique sampling stations and 2 samples were replicates from the same sampling station). Two asterisks in “n per year” column indicate that individuals were pooled from multiple sampling stations within the same sampling area to achieve a suitable number of individuals for biochemical analysis (e.g., “1, 2**” indicates that 1 sample was from a single unique sampling station, and 2 samples were from pooled individuals from multiple sampling stations). Where no asterisks are present, n per year reflects unique stations sampled. Full station and sampling information is reported in the dataset.
